# Mini Review: Risk Assessment, Clinical Manifestation, Prediction, and Prognosis of Mucormycosis: Implications for Pathogen- and Human-Derived Biomarkers

**DOI:** 10.3389/fmicb.2022.895989

**Published:** 2022-06-20

**Authors:** Jaime David Acosta-España, Kerstin Voigt

**Affiliations:** ^1^Jena Microbial Resource Collection, Leibniz Institute for Natural Product Research and Infection Biology – Hans Knöll Institute, Jena, Germany; ^2^Institute of Microbiology, Friedrich Schiller University Jena, Jena, Germany; ^3^Department of Medical Microbiology, Hospital Vozandes Quito, Quito, Ecuador; ^4^School of Medicine, Universidad de las Américas, Quito, Ecuador

**Keywords:** fungal infection, immunocompromised patients, COVID-19-associated mucormycosis, host markers, host genetics, assessment, prognosis

## Abstract

Mucormycosis is a fungal disease caused by members of the fungal order Mucorales, which are abundantly found in terrestrial environments. The fungi propagate clonally *via* mitospores, which are transmitted to humans through the air and cause superficial or invasive infections. The disease has emerged in recent years and coincides generally with immunosuppression on the patient side. Mucormycosis is still rarely recognized in the clinical because of its unspecific symptoms which often triggers misdiagnosis with bacterial or viral infections leading to prolonged therapeutic cycles and loss of valuable time to manage mucormycosis properly. Infected patients develop various clinical forms, most notably ranging from rhinocerebral *via* pulmonary to gastrointestinal forms. Traditional diagnosis is based on culture and histopathologic examinations of the affected tissue. But, the achievement of a precise result is time-consuming, labor-intensive, requires mycological expertise and the finding appears often too late. A rapid and precise diagnosis is mandatory because symptoms are non-specific and the disease is rapidly progressing with often fatal outcome. Mucormycosis was increasingly associated with other infections and underlying conditions and risk factors causing comorbidities, which are difficult to successfully manage. This mini-review summarizes the current knowledge on the epidemiology and causative agents of mucormycosis, transmission, risk factors, clinical presentation, diagnosis, and highlights the lack of appropriate biomarkers on the pathogen and the host sides for rapid pathogen and host susceptibility detection, respectively. Fungal antigens and single nucleotide polymorphisms (SNPs) in human host genes are useful for the assessment of susceptibility. This mini-review addresses possibilities for early prediction of susceptibility to mucormycosis based on forecasting of the risk of infection with fungal pathogens other than Mucorales. The topic of early prediction and diagnosis of mucormycosis represents a current research gap and highlights the importance of potential future developments in the area of risk assessment, susceptibility prognosis in conjunction with early diagnosis to reduce mortality in patients suffering from mucormycosis.

## Highlights

– Summary of the biomarkers currently available for the diagnosis of mucormycosis.
– Overview of predisposing factors and comorbidities from a medical point of view.
– Encourage the exploration of novel biomarkers for pathogen and host susceptibility detection.

## Introduction

Mucormycosis (formerly: zygomycosis) is a group of diseases affecting various anatomical sites. The infections are caused by filamentous fungi of the order Mucorales, a dominant group among zygosporic fungi which were formerly summarized as zygomycetes. The fungi are ubiquitous and predominate in the decomposition of organic matter. Among the clinically important genera (1) *Rhizopus*, (2) *Lichtheimia*, (3) *Mucor*, (4) *Rhizomucor*, (5) *Thermomucor*, (6) *Syncephalastrum*, (7) *Cunninghamella*, (8) *Cokeromyces*, (9) *Apophysomyces* and (10) *Saksenaea*, we can predominantly observe the following species: (1) *Rhizopus arrhizus* (formerly: *R. oryzae*) and *R. microsporus*, (2) *Lichtheimia corymbifera* (formerly: *Absidia corymbifera*), *L. ornata, L. ramosa*, (3) *Mucor circinelloides, M. lusitanicus*, *M. ramosissimus, M. racemosus, M. hiemalis*, (4) *Rhizomucor miehei* and *Rh. pusillus*, (5) *Thermomucor indicae-seudaticae*, (6) *Syncephalastrum monosporum* and *S. racemosum*, (7) *Cunninghamella elegans* (formerly: *C. bertholletiae*), (8) *Cokeromyces recurvatus*, (9) *Apophysomyces elegans* and *A. variabilis*, and (10) *Saksenaea vasiformis* (Ribes et al., 2000; Roden et al., 2005; Spellberg et al., 2005; Petrikkos et al., 2012; Hassan and Voigt, 2019; de et al., 2020; Index Fungorum, 2022).

**FIGURE 1:**
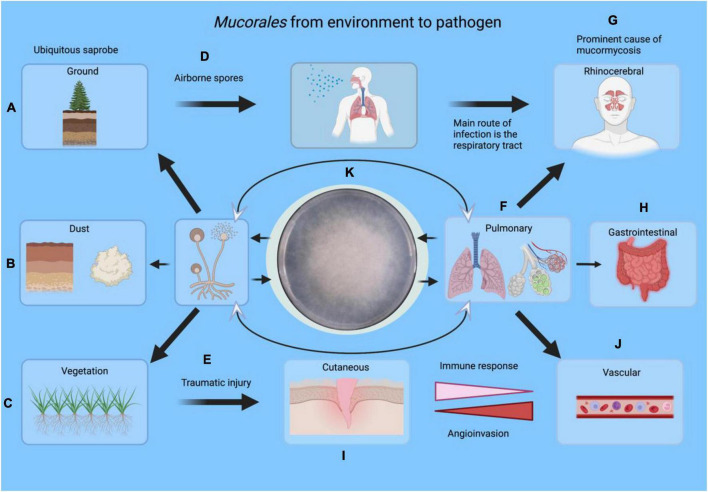
An overview of infection routes of Mucorales from the environment toward the development as pathogens. Mucorales can be found in soil **(A)**, dust **(B)**, and vegetation **(C)**. The spores can infect humans through the respiratory tract *via* airborne spores **(D)**, injuries **(E)**, pulmonary **(F)** rhinocerebral **(G)** or the gastrointestinal tract **(H)**. Cutaneous cases, on the other hand, are associated with vegetal trauma or motor vehicle accidents **(I)**. Any of these local infections can cause vascular spread leading to thrombosis **(J)**. Specimens **(F–I)** can be obtained for laboratory diagnosis by culture **(K)** or other methods. Eventually, the fungus returns to the environment where it belongs. This figure was created in BioRender.com.

Although inhalation of spores is the most common route of transmission, the disease can also be acquired cutaneously and gastrointestinally (Figure 1). This life-threatening disease primarily affects immunosuppressed, diabetic and all types of immunocompromised patients, which suffer from a primary bacterial or fungal infection (Choi et al., 2019). Most actually, mucormycosis associated with Coronavirus disease 2019 (COVID-19) became a new threat due to high-dose corticosteroid therapy during the SARS-CoV-2 pandemic [as reviewed by Hoenigl et al. (2022)]. Diagnosis in patients with mucormycosis is complicated and delayed. Treatment is based on surgical debridement of the affected areas and antifungal therapy, which has low penetrance, explained by the large areas of necrosis that these infected patients develop in association with angioinvasion (Farmakiotis and Kontoyiannis, 2016; Patel et al., 2020; Pal et al., 2021).

This mini-review will outline the various fields of research targeting risk assessment, prediction, and prognosis based on human-derived biomarkers and their variants.

## Risk Assessment

In patients with mucormycosis, cellular immunity has been shown to be critical in combating these filamentous fungi. Therefore, comorbidities or diseases that compromise immunity and surface barrier integrity count as risk factors for acquiring the infection. The study by Jeong et al. (2019) showed that the following comorbidities were associated with a higher percentage of mucormycosis: diabetes mellitus, hematologic malignancy, diabetic ketoacidosis, acute myeloid and lymphoblastic leukemia, lymphoma, hematopoietic stem cell transplantation, and others (Ghuman and Voelz, 2017).

On the other hand, in patients without comorbidities, the main risk factors were shown in the population that used voriconazole, suffered injection sites, had a car accident, used corticosteroids, used fluconazole, had neutropenia, among others (Jeong et al., 2019). Increased mortality was observed in patients with diabetes mellitus and immunocompromised states in a follow-up 180 days after their diagnosis (Hong et al., 2013).

COVID-19 patients suffering from mucormycosis have raised the alarm worldwide, with a focus on India. Indeed, it is important to note that India already had a 70-fold higher risk of mucormycosis compared with developed countries. Despite this, an Indian multicenter epidemiologic study reported a two-fold increase in mucormycosis cases in 2019 compared with 2020 (Banerjee et al., 2021; Mathew et al., 2021; Pal et al., 2021; Rocha et al., 2021). The risk factors for COVID-19 associated mucormycosis (CAM) highlighted in the aforementioned studies are: cytokine storm (interleukin 6), islet damage, elevated ferritin, and endotheliitis. It is important to note that it is likely that the sum of cytokine storm, ketoacidosis, and uncontrolled corticosteroid use increases the risk of hyperglycemia and impaired phagocytosis, leaving patients susceptible to CAM (Mathew et al., 2021; Pal et al., 2021). These and other hypotheses are being studied to understand the increase in cases of mucormycosis in patients infected by SARS-CoV-2. A comparison between the risk factors in 3 studies in the general population and in a study in patients with COVID-19 is shown in Table 1.

**TABLE 1:** Comparison of underlying conditions and predisposing factors for the development of mucormycosis.

	General population			COVID-19 associated
		
Risk factors	Jeong et al., 2019	Patel et al., 2020	Prakash et al., 2019	Hoenigl et al., 2022
Diabetes mellitus	40% (340)	74% (465)	91% (172)	83% (80)
Diabetic ketoacidosis	21% (71)	50% (465)	90% (31)	49% (80)
Hematological malignancy	33% (275)	1.3% (465)	84% (19)	6% (80)
Corticosteroid use	33% (273)	3.7% (465)	90% (27)	79% (80)
Motor vehicle accident	33% (28)	–	–	–
Neutropenia	20% (169)	–	89% (16)	–
Injection sites	42% (34)	–	–	–
Use of cancer chemotherapy	18% (149)	–	87% (16)	–
Use of calcineurin inhibitors	16% (133)	–	–	–
Voriconazole	52% (48)	–	–	–
Fluconazole	25% (23)	–	–	–
Other minor injury	14% (12)	–	–	–
Cuts/grazes	14% (11)	–	–	–
Other open wound trauma	21% (18)	6.9% (465)	37% (31)	–

*The percentages indicate the proportion of patients affected by the particular risk factor developed mucormycosis in each population.*

*The number in brackets indicate the total number of patients were affected by the particular risk factor.*

*“–” indicates that no data were found.*

## Physical Examination

Regarding its anatomical localization, mucormycosis is divided into six forms: (1) rhinocerebral, (2) pulmonary, (3) cutaneous, (4) gastrointestinal, (5) disseminated, and (6) uncommon forms. The appearance and clinical manifestations vary according to this classification and are described below (Jeong et al., 2019).

### Rhinocerebral Disease

The patients in whom the rhinocerebral variant occurs are usually diabetics and/or patients with ketoacidosis. This variant is characterized by unilateral involvement accompanied by facial pain, retro-orbital headache, fever, hyposmia, numbness, and nasal congestion. Within a few days, this condition develops into a black discharge. Later, invasion of orbital nerves and vessels may be observed, including diplopia and loss of vision, nasal cavity, palate, and face with black eschar, among others. The neurological presentation may include brain involvement and loss of consciousness, which unfortunately means a poor prognosis (Szalai et al., 2006).

### Pulmonary Disease

This pulmonary presentation is common in patients with hematologic malignancies and a history of neutropenia. Concurrent sinusitis and pulmonary disease may be expected. Pulmonary infection presents with non-specific symptoms of cough, fever, dyspnea, and hemoptysis in patients with necrosis. Auscultation reveals rales and a decreased vesicular murmur. The infection may even be so aggressive that it exceeds the values for mortality compared with other infectious agents. Cellulitis of the affected chest wall may occur in these patients (Peng et al., 2019).

### Cutaneous Disease

Skin diseases are often associated with trauma and loss of the skin barrier. Cases have even been reported in association with biomedical devices such as catheters, insulin use, etc. Initially, it manifests as cellulitis that progresses to necrosis of the affected tissue with the formation of a black scab. This necrosis, as in other manifestations, is the expression of vascular invasion that clogs blood vessels and leads to tissue damage (Rrapi et al., 2021).

### Gastrointestinal Disease

Gastrointestinal infections occur in the severely malnourished patients undergoing organ transplantation. They usually involve mainly the stomach, ileum, colon, and liver. The clinical manifestations are non-specific, characterized by abdominal pain, nausea, vomiting, abdominal distension, and may even present with hematochezia or obstruction. In case of rupture of intestinal integrity, patients present symptoms of peritonitis (Spellberg, 2012; Wotiye et al., 2020).

### Disseminated Disease and Uncommon Presentations

To a lesser extent, disseminated forms of the disease may also occur, affecting the kidneys, heart, and other organs. The symptoms are related to the site of infection (Devana et al., 2016; Krishnappa et al., 2019).

## Diagnostic Methods, Pathogen, and Human-Derived Biomarkers

### Diagnostic Methods

Clinical criteria, risk factors, histopathologic findings, cultures, and imaging studies should be considered in the diagnosis of mucormycosis, especially in health centers treating patients with COVID-19. For direct microscopic, cultural, and histopathologic analysis, it is advisable to obtain a sample of the affected tissue, preferably by biopsy, although fine-needle aspiration is also possible. Histopathological examination of tissue infected with Mucorales shows damage to the tissue and invasion of the blood vessels, accompanied by the presence of hyphae with the following characteristics:

• Broad, pauciseptate hyphae.
• Coenocytic to irregular branching of hyphae.
• Large branching angle, approximately rectangular.

Fungal culture has a variable sensitivity for diagnosis, it is advisable to send a biopsy fragment of the infected tissue. Unfortunately, the yield of mucormycosis pathogens is low, and these microorganisms may also have slow growth. Another disadvantage of biopsy is that more invasive endoscopic procedures may be required to obtain tissue, especially for infections of the lung and gastrointestinal tract, to name a few.

Complementary laboratory tests may be useful and may reveal neutropenia in the white blood count, low blood pH in diabetic ketoacidosis, and positive urinary ketone levels in diabetic ketoacidosis. Imaging studies can aid in diagnosis, although it should be noted that computed tomography (CT) of the sinuses is less sensitive than magnetic resonance imaging (MRI) for detecting soft tissue invasion. CT thoracic examination with intravenous contrast is a sensitive test for detecting abnormalities in pulmonary mucormycosis, and CT of the brain may not be able to distinguish between abscesses and early infarcts (Gamba et al., 1986; McAdams et al., 1997; Kontoyiannis and Lewis, 2006; Turunc et al., 2008).

Laboratory and imaging studies are helpful, but they are not foolproof, as other pathologies may yield similar results. Early diagnosis is critical for treatment and improving prognosis. Biomarkers have been poorly studied and lack clinically approved options. Therefore, early diagnosis and monitoring of mucormycosis by detection of circulating DNA in serum is mandatory to manage the disease. A cohort study of a total 44 patients described the detection of circulating DNA in one patient with mucormycosis, which demonstrated the diagnostic utility and the accurate quantification of the fungal DNA load enabling therapeutic monitoring (Millon et al., 2016).

In 2014, a study was published on serial monitoring of mucoralean DNA load in serum samples from a patient with disseminated mucormycosis. The qPCR assay was used to detect circulating DNA of *Mucor*/*Rhizopus*, *Lichtheimia*, or *Rhizomucor*, which showed very high variability and was detectable from day 9 of infection, with elevation peaks at days 15 and 38 (Shigemura et al., 2014). These results contrast with the case report of detection of the circulating fungal DNA by polymerase chain reaction in a fatal case of infection with *Cunninghamella elegans* (synonym: *C. bertholletiae*), in which detection of *Cunninghamella bertholletiae* DNA in serum was highest on day 1 at 18.0 copies/ml and on day 4 (101.0 copies/ml) (Hiramoto et al., 2020).

Especially in the context COVID-19 of evaluating hospital trends in mucormycosis and other fungal infections, this may become essential today. An evaluation of trends for all fungal infections should be considered which uses denominator data to calculate incidence and seasonality. It is recommended that hospitals review 12- to 24-month back microbiologic cultures and histopathologic specimens with evidence of tissue invasion by fungal hyphae to obtain an epidemiologic curve and associated hospital services or areas. If mucormycosis outbreaks are suspected, the environment should also be reviewed through a general inspection for mold, leaks, dirty HVAC systems, cleaning of the environment, construction and maintenance areas, indoor air temperature, and humidity records, including any days when humidity exceeded 60%, dates of air filter changes, etc (Hartnett et al., 2019).

### Pathogen and Human-Derived Biomarkers

Invasive fungal disease is a challenge for medicine, and a rapid diagnosis can prevent tissue damage and complications and reduce mortality. Currently available biomarkers for rapid detection of fungal infections include carbohydrates derived from the cell wall of fungi, such as galactomannan (*Aspergillus*, *Penicillium*, *Paracoccidioides*, *Histoplasma*, *Fonsecaea*, and *Cryptococcus*). (1→3)-β-D-glucan (*Aspergillus* spp., *Candida* spp., *Fusarium* spp., *Trichosporon* spp., *Saccharomyces cerevisiae*, *Acremonium* spp., *Coccidioides immitis*, *Histoplasma capsulatum*, *Sporothrix schenckii*, and *Pneumocystis jirovecii*). *Candida* mannan (*C. albicans*, *C. glabrata*, and *C. tropicalis*) (Huppler et al., 2017).

It is important to remember that the galactomannan levels are low in mucormycosis because Mucorales do not expose gluconic cell wall sugars on the surface of their hyphae (Pickering et al., 2005). It has been used with an approach to aspergillosis that showed sensitivity between 60 and 80% in hematologically neutropenic patients (Pfeiffer et al., 2006; Lamoth, 2016). Detection of dihexasaccharide in serum (MS-DS) was associated with mucormycosis in 9 of 10 patients using matrix-assisted laser desorption/ionization time-of-flight mass spectrometry (MALDI-TOF) (Mercier et al., 2018).

*In vitro* studies of specific T cells in Mucorales-infected patients could be detected only in patients with invasive mucormycosis which represented elevated production of IL-4, IFN-γ, IL-10, and to a lesser extent IL-17 and belonged to CD4+ subsets or CD8+. Nevertheless, CD8 + Mucorales-specific T cells can produce either IL-4 or IL-10, predominantly in the late phase of infection. These studies should be recruited in a larger population to demonstrate their clinical utility in diagnosing patients, especially in early forms of the disease (Potenza et al., 2011).

The presence of the cell wall carbohydrate fucomannan has been studied as a biomarker in invasive mucormycosis in mice (Orne et al., 2018). During infection, this biomarker is secreted in blood, urine, serum, and bronchoalveolar lavage fluid (BALF). A lateral flow assay (LFA) for the detection of fucomannan was developed (mAb 2DA6). BALF, serum, and urine were collected 3–4 days after intratracheal infection of immunosuppressed or DKA mice with spores of various Mucorales, including *Rhizopus arrhizus* (synonym: *R. delemar* and *R. oryzae)*, *L. corymbifera*, *M. circinelloides* and *C. elegans* (*synonym: C bertholletiae*). Samples collected 3 and 4 days post-infection were positive, demonstrating the ability of LFA to provide early positive results. The highest reactivity was observed in urine samples (Orne et al., 2018).

Another recently described possibility is the detection of *Rhizopus*-specific antigen (RSA). For this purpose, a sandwich enzyme-linked immunosorbent assay (ELISA) was developed to detect RSA levels in the serum of vaccinated mice. This proved to be of interest as RSA levels were higher in mice with mucormycosis (15.1 ng/ml) than in mice with aspergillosis (0.53 ng/ml), the latter having levels close to the negative control (0.49 ng/ml) (Shibata et al., 2020).

## Current Aspects in Diagnosis and Prediction of other Fungal Diseases and their Implications for Novel Diagnostic Strategies for Mucormycosis

### From the Pathogen Side: Ligands, Pathogen-Associated Molecular Patterns

Homologs of the spore-coat protein CotH are widespread in Mucorales and absent in non-invasive species (Gebremariam et al., 2014). Thus, CotH is a promising target for the early diagnosis of mucormycosis (Baldin et al., 2018). However, a Mucorales-specific marker may neglect concomitant fungal opportunists, as is the case with pioneering invasive mucormycosis, which usually follows invasive aspergillosis. Patients with aspergilloma and invasive aspergillosis develop an antibody response to *Aspergillus fumigatus* mannoprotein 1 (AFMP1), suggesting that the protein is a target for host humoral immunity (Yuen et al., 2001). Indeed, clinical evaluation revealed that an enzyme-linked immunosorbent assay-based antibody (ELISA) test using the AFMP1 was 100% sensitive for patients with aspergilloma and 33.3% sensitive for patients with invasive aspergillosis (Chan et al., 2002).

For this reason, it is important to analyze surface proteins and even polysaccharides that can be examined in clinical specimens for early detection of infectious Mucorales.

### From the Host Side: Receptors, Pattern Recognition Receptors

The endothelial cell receptor GRP78 is required for the pathogenesis of mucormycosis in diabetic mice (Liu et al., 2010). Another target could be Pentraxin 3 (PTX3), which has been shown to be a promising marker for aspergillosis (Cunha et al., 2014). Mucormycosis frequently co-occurs with aspergillosis (Alfano et al., 2006; Tsikala-Vafea et al., 2020; Johnson et al., 2021; Zayet et al., 2021). SNPs in PRRs like Toll-like receptors (TLRs) have already been associated with increased susceptibility to fungal infections (Bergantim et al., 2013; Wang et al., 2013; Fischer et al., 2016; Zhang et al., 2019; Aqsa et al., 2020). The TLR2 SNP rs5743708 (R753Q, GA/AA genotype, *n* = 12) is associated with a higher risk of pneumonia and invasive fungal infections in patients with acute myeloid leukemia undergoing chemotherapy (Fischer et al., 2016). TLR with SNP’s have previously been associated with increased susceptibility to fungal infections (Wang et al., 2013; Fischer et al., 2016). TLR2 SNP rs5743708 (R753Q, GA/AA genotype, *n* = 12) is associated with a higher risk of pneumonia and invasive fungal infections in patients with acute myeloid leukemia receiving chemotherapy (Fischer et al., 2016).

No studies were found analyzing genetic variations that increase host risk for developing mucormycosis. The discovery of these markers in other diseases such as invasive aspergillosis leads us to hypothesize that this may also be the case in these patients, especially since there may be individual susceptibility, as not all patients with diabetic ketoacidosis or hematologic malignancies are infected (Naik et al., 2021).

## Conclusion

Mucormycosis is a rare disease with a high-mortality potential. Several risk factors for the acquisition of mucoralean infections have been described, including a decrease in cellular immunity and disruption of anatomic barriers. Among the most important are diabetic patients, especially with ketoacidosis, malignancies, trauma, neutropenia, use of voriconazole, fluconazole, and corticosteroids. It is important to discuss the differences in prevalence in different regions, which are likely due to population numbers, environmental conditions, control and prevalence of comorbidities such as diabetes, immunity in oncologic diseases, and even underdiagnosis of patients with mucormycosis in each country, among other factors and underlying comorbidities.

In 2020 and 2021, superinfection by mucormycosis raised alarm in patients with COVID-19, although this association is still under investigation, with a significant prevalence in India that is multifactorial. Cytokine storm (IL-6), islet cell damage, elevated ferritin, and corticosteroid use in mainly patients with diabetes patients have led to a doubling of mucormycosis cases in India compared to 2019 and 2020. There are several clinical presentations in these patients, and the rhinocerebral form clearly predominates, although almost any organ can be affected in patients with risk factors. Diagnosis is complex, and late effects, risk factors, clinical presentation, histologic criteria, culture, molecular evidence, imaging, and laboratory testing must be considered. Currently, isolation of the fungus in an axenic culture and detection of fungal structures in histopathology are used to confirm infection. However, it is important to keep in mind that not all hospitals have staff trained in mycological diagnosis and do not have a budget for molecular detection of associated microorganisms, especially in low-resource countries, so biomarkers may be a more viable option for rapid diagnosis.

In patients infected with Mucorales, time is a valuable commodity to reduce mortality. Therefore, SNPs could be of great help in determining which patients are more susceptible to mucormycosis due to mutations in TLRs. Another option could be the detection of early-emerging antigens, such as CotH or Fucomannan. Both detection of SNPs in TLRs and detection of antigens that could be a predictive factor and/or early diagnosis, especially in patients with hematologic and oncologic diseases, diabetics with ketoacidosis, and other high-risk groups. However, these possibilities should be explored in the future. Finally, epidemiologic surveillance and investigation of suspected outbreaks of mucormycosis in health care settings have again become very important, especially because of the increase in COVID-19 associated mucormycosis.

## Author Contributions

JA-E and KV drafted and revised the manuscript. Both authors contributed to the article and approved the submitted version.

## Conflict of Interest

The authors declare that the research was conducted in the absence of any commercial or financial relationships that could be construed as a potential conflict of interest.

## Publisher’s Note

All claims expressed in this article are solely those of the authors and do not necessarily represent those of their affiliated organizations, or those of the publisher, the editors and the reviewers. Any product that may be evaluated in this article, or claim that may be made by its manufacturer, is not guaranteed or endorsed by the publisher.
